# Research, Modelling and Prediction of the Influence of Technological Parameters on the Selected 3D Roughness Parameters, as Well as Temperature, Shape and Geometry of Chips in Milling AZ91D Alloy

**DOI:** 10.3390/ma15124277

**Published:** 2022-06-16

**Authors:** Monika Kulisz, Ireneusz Zagórski, Jerzy Józwik, Jarosław Korpysa

**Affiliations:** 1 Department of Organisation of Enterprise, Management Faculty, Lublin University of Technology, 20-618 Lublin, Poland; 2 Department of Production Engineering, Mechanical Engineering Faculty, Lublin University of Technology, 20-618 Lublin, Poland; i.zagorski@pollub.pl (I.Z.); j.jozwik@pollub.pl (J.J.); j.korpysa@pollub.pl (J.K.)

**Keywords:** magnesium alloy, rough milling, roughness 3D parameters, chip temperature, chip geometry, artificial neural networks

## Abstract

The main purpose of the study was to define the machining conditions that ensure the best quality of the machined surface, low chip temperature in the cutting zone and favourable geometric features of chips when using monolithic two-teeth cutters made of HSS Co steel by PRECITOOL. As the subject of the research, samples with a predetermined geometry, made of AZ91D alloy, were selected. The rough milling process was performed on a DMU 65 MonoBlock vertical milling centre. The machinability of AZ91D magnesium alloy was analysed by determining machinability indices such as: 3D roughness parameters, chip temperature, chip shape and geometry. An increase in the feed per tooth f_z_ and depth of cut a_p_ parameters in most cases resulted in an increase in the values of the 3D surface roughness parameters. Increasing the analysed machining parameters did not significantly increase the instantaneous chip temperature. Chip ignition was not observed for the current cutting conditions. The conducted research proved that for the adopted conditions of machining, the chip temperature did not exceed the auto-ignition temperature. Modelling of cause-and-effect relationships between the variable technological parameters of machining f_z_ and a_p_ and the temperature in the cutting zone T, the spatial geometric structure of the 3D surface “Sa” and kurtosis “Sku” was performed with the use of artificial neural network modelling. During the simulation, MLP and RBF networks, various functions of neuron activation and various learning algorithms were used. The analysis of the obtained modelling results and the selection of the most appropriate network were performed on the basis of the quality of the learning and validation, as well as learning and validation error indices. It was shown that in the case of the analysed 3D roughness parameters (Sa and Sku), a better result was obtained for the MLP network, and in the case of maximum temperature, for the RBF network.

## 1. Introduction

Very often, in the research on the machinability of magnesium alloys, monolithic tools with sintered carbide inserts or tools with polycrystalline diamond (PCD) inserts are used. These tools are often used in the production of light alloy parts, including parts made of magnesium alloys in engineering, automotive and aviation industries, as well as for scientific research purposes. An interesting issue seems to be the use of tools made of high speed steel (HSS). The unit cost of machining using HSS cutters is small compared to sintered carbide or very expensive PCD inserts.

### 1.1. Surface Roughness Parameters Evaluating of Magnesium Alloys after Cutiig Processes

The quality and roughness of the surface is the key manufacturing index in the production of various parts of various materials, both in the automotive and aerospace industries. These requirements are increasing, especially when mating of two or more elements in an assembly is critical. It is also an interesting scientific aspect during the execution of machining processes. For magnesium alloys, the roughness obtained, as well as the general manufacturing accuracy (with a relatively small spread of dimensional values for high manufacturing precision classes), can be successfully comparable or even better than after finish machining (Ra ≤ 0.16 µm [1], tolerance classes IT2–IT5 [2]). In research works, the so-called basic parameters of surface roughness are analysed, while a much broader approach is required for a comprehensive assessment, taking into account, for example, surface geometry features, such as skewness and kurtosis.

**Figure 1:**
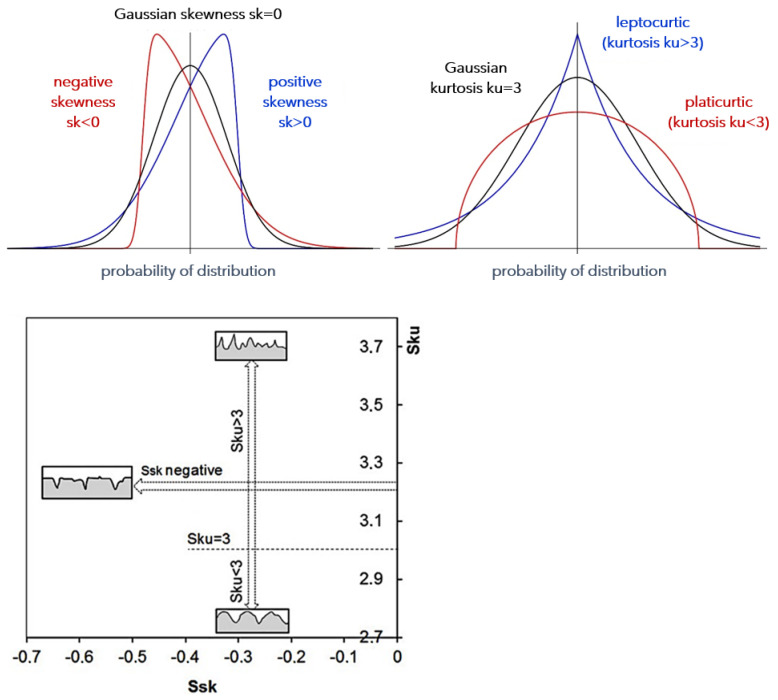
Skewness and kurtosis, as well as graphical interpretation related to the surface geometrical structure and its parameters Ssk and Sku [3].

The skewness coefficient Ssk is a measure of asymmetry of a surface. The skewness coefficient Ssk > 0 indicates the right-hand asymmetry of the distribution, Ssk < 0 indicates the left-hand asymmetry of the distribution and Ssk = 0 indicates a symmetrical distribution. Kurtosis Sku is a measure of the concentration of results, a measure of location. This measure indicates how many results/observations are similar the mean value and if most of the observed results have a value similar to the mean value. Kurtosis is a measure of flattening of the surface compared to the normal distribution and its characteristic value is Sku = 3 (Figure 1). Sku values < 3 mean a distribution relatively flatter than the Gaussian distribution, the surface has more rounded peaks, while Sku values > 3 mean a more convex distribution, the surface has sharper peaks [3].

An interesting aspect is also the use of Mg-Ca0.8 [4,5,6] and Mg-Ca1.0 [7] alloys, used as biocompatible components in medicine. In milling using a face milling cutter with PCD inserts, an average value of the Ra parameter was obtained in the range of approximately 0.5 µm [4] and approximately 0.4 µm for low dry milling and low plasticity burnishing [5]. However, in milling using a face milling cutter with sintered carbide inserts [6], lower values of the roughness parameter Ra = approximately 0.09–0.8 μm were obtained in inverse milling, while in conventional milling, the roughness was Ra = approximately 0.9–1.4 μm. However, in milling using a face milling cutter with the so-called diamond-like coating (DLC) inserts [7], Ra = approximately 0.08–0.16 μm was obtained.

In the work [8], when milling AZ31B alloy and cooling the cutting zone with compressed air, it was observed that the surface roughness (Ra parameter) increased when the feed and number of teeth is increased. However, in terms of changes to v_c_, the roughness remains almost unchanged. Therefore, it is recommended to reduce the number of teeth to the minimum necessary. A face milling cutter with uncoated sintered carbide inserts was used in this work.

In the work [9], AM60 alloy was milled to find the optimal combination of cutting parameters (lowest roughness of surface), obtaining Ra = approximately 0.3 µm. When machining AZ61 alloy [10], the surface roughness defined with the Ra parameter was within the range of approximately 0.1–0.4 µm. Milling was carried out using a face milling cutter with sintered carbide inserts. Similarly, in the work [11], AZ61 alloy was machined using a face milling cutter with sintered carbide inserts. The feed per tooth parameter and the change of the cutting direction had the most significant influence on the surface roughness. Changes of roughness caused by increasing v_c_ and a_p_ were minor. The values of the Sa parameter obtained for direct feed were approximately 0.14–0.8 μm, while for reversed feed, the values were approximately 0.2–0.7 μm. It was observed that the spread of values of Sa were narrower using MQL cooling than during dry milling.

In the work [12], it was proved that with the use of low feed values of (0.03–0.09 mm/tooth) and small depths of cut of (0.2–0.3 mm), it is possible to obtain a very low surface roughness Ra = approximately 0.06–0.13 µm. The frequently used AZ91D alloy was also used for this test. In this case, the lowest Ra value was achieved at the lowest of the tested cutting speeds (v_c_ = 900 m/min). The obtained test results proved that it is possible to eliminate grinding or polishing in favour of face milling as the finish machining operation.

Similarly, in the work [13], a cutter with uncoated sintered carbide inserts and AZ91D alloy were used. All milling tests were carried out on down milling under dry condition. The machining was carried out with a speed of up to 400 m/min (conventional speed). The Ra parameter increased with an increase of v_c_ and f_z_. The lowest value of Ra was obtained when using the low cutting speed v_c_ = 50 m/min and low feed f_z_ = 0.2 mm/tooth (Ra approximately 0.5 µm).

In scientific tests, uncoated sintered carbide inserts [14]; inserts with TiN- [9], TiAlN- [15], TiB2- [2] or TiAlCN-type protective coatings; and high-quality polycrystalline diamond inserts are often used [1]. For example, it is possible to rough machine with machining parameters defined as effective (v_c_ = 1200 m/min, f_z_ = 0.15 mm/tooth, a_p_ = 6 mm) and reach the roughness parameter Ra (measured on the face of the sample) at the level of approximately 0.5 µm (at γ = 5°) and approximately 2 µm (at γ = 30°). The mean value of the Ra parameter (measured this time on the side wall of the sample) was lower and amounted to approximately 0.3 µm for the given conditions [15]. 

### 1.2. Temperature in the Cutting Zone

For the sake of safety, the key issue is assessment of the temperature during machining of magnesium alloys. The conducted research on various types of magnesium alloys proves that it is possible to mill without increasing the risk of ignition of chips formed during the process of machining. Generally, it can be stated that the fire ignition of chips could happen when the cutting temperature is close to the melting point of magnesium during high-speed cutting. An experimental study [16] shows the mean temperature on the flank face in high-speed dry cutting of AZ91 magnesium alloy. Foreign thermocouples (type k) placed in the workpiece were used in the tests. In the HSC process, the mean flank temperature is less than that on the rake face (cutting temperature). The undeformed chip thickness is very thin and of the same order of cutting edge radius. The mean flank temperatures in various cutting conditions were measured and the collected chips were examined under SEM to find the burn marks. It was found that below the mean flank temperature of 302 °C, there were no burn marks on the chips (at the cutting speed of 816 m/min and at undeformed chip thickness 9 µm). The mean flank temperature can reasonably be used to predict the occurrence of fire in high-speed cutting of magnesium alloys. The temperature increases with an increase in cutting speed, and increases dramatically with a decrease in the undeformed chip thickness.

In the work [4] the temperature in the cutting zone was also analysed and determined both in the tool/chip interfaces (across chip) and in the subsurface sections. The analyses and simulations performed show that the predicted temperatures at the tool/chip interfaces were close to the melting temperature of the Mg-Ca0.8 alloy (approximately 600 °C). Similarly, in [17], the temperature in the cutting zone that would be generated during milling the Mg-Ca0.8 alloy was analysed. A total of five simulation cases have been performed covering five cutting speeds. The authors point out that when analysing temperature distributions, two aspects should be taken into account: the size of the area covered by a certain temperature and the temperature itself. Although temperatures close to 600 °C were observed in the simulation, it should be noted that there would probably be no spontaneous combustion during the treatment as the predicted temperatures would be the upper bound of machining temperatures in practice under the cutting conditions (heat conductions between the tool/workpiece and workpiece/environment were not incorporated so it implies that material melting and chip ignition would not happen for the concerned machining conditions). In addition, the temperature in almost the entire volume of the chip is approximately 150–450 °C, so it is highly probable that there will be no chip ignition and no fire hazard, as the chips can only ignite if the melting point (estimated at 516.6 °C) is exceeded in the entire chip volume. This is confirmed experimentally since no spark or chip ignition occurred during milling.

In the work [18], the mean flank temperature was measured (through mounting two K-type thermocouples). Magnesium alloy AM50A was used. The effect of the cutting speed on the temperature rise of the tool flank was analysed. It was shown that the temperature first increases and then decreases as the cutting speed increases, whereas, in [19], chip ignition attempts were made during the machining of AM50A and AZ91D magnesium alloys. The effect of the cutting parameters on the ignition of chips during face milling was analysed. It is interesting to find that AZ91D is more inflammable than AM50A in dry face milling. 

Similarly, in the work [20], the temperature distribution in high-speed milling was analysed for the AZ91C alloy. The temperature distribution was measured using an infra-red thermometer (machining zone temperature) and the contact method (platinum temperature sensors for the work-piece temperature). The results show that the work-piece temperature is reduced as the cutting speed passes the cutting speed of 452 m/min in high-speed milling, while the machining zone temperature is increased as a result of the increase in the cutting speed. No ignition was observed during the machining and in any of the given range of machining parameters.

In [21], the typical problems that occur when measuring the temperature in the cutting zone during milling of metal alloys were discussed. Selected results of temperature measurements with the use of three measurement methods were also presented: the k type foreign thermocouple, optical pyrometry and thermal imaging camera, whereas, in [22], the results of the measurement of chip temperature in the cutting zone with the use of sintered inserts with a TiAlN coating and AZ31 and AZ91HP magnesium alloys are presented in more detail. The influence of the machining parameters on the maximum chip temperature in the cutting zone during dry milling was analysed. It was observed that the chip temperature in the cutting area is substantially lower than the temperature needed for chip ignition or the melting point of magnesium alloys, whereas, in [23], the so-called time to ignition, ignition temperature and chip morphology after milling with TiAlN-coated sintered carbide inserts were analysed. Additionally, the successive stages preceding chip ignition for magnesium alloys (AZ31 and AZ91HP) were presented. Whereas, in the work [24], the chip temperature in the cutting zone was also analysed when milling the mentioned magnesium alloys, in this case, a high-quality cutter with polycrystalline diamond PCD inserts was used as the tool, for example, chip fractions and the mass of chips after machining (taking into account different chip fractions). The work presents chip temperature results in the form of box-plot charts and a summary table covering various temperature values (including the entrance and the exit of the workpiece, including the so-called “outliers” which differ from other measured values, including the stable region only). In addition, metallographic photographs of magnesium alloy chips and the so-called “cauliflowers” area in the magnesium chips ignited on a heating plate of a specially designed test stand were presented. 

Unfortunately, research works are often performed using the conditions of machining parameters (mainly depth of cut) that are unlikely to be implemented in the common industrial practice. The milling tests using small cut depths of a dozen or so micrometres [25] are, of course, valuable scientific works. In terms of industrial applications, the tests carried out in machining conditions similar to those used in industry are valuable, if performed in an effective and efficient manner. 

Alternative methods of describing the complexity of the analysed surface are, inter alia, multi-scale methods. Classical methods decompose the measured surface topography into basic factors, e.g., roughness or waviness. On the other hand, multiscale methods allow for a more comprehensive description of the surface topography [26]. Structure analysis can be performed using, inter alia, wavelet transformation, which was argued by Sun et al. in his research [27], who based on the signals obtained during measurements, assessed the surface structure or Gogolewski when determining the minimum chip thickness [28].

### 1.3. Chip Shape and Geometry

Chips, often treated only as a waste effect of the machining process, can also provide valuable information, such as the temperature in the cutting zone, for example. Some features of chips may also indicate that the process of machining is performed in safe manner. In the work [4], the characteristics and morphology of chips obtained during milling of the Mg-Ca0.8 alloy with the use of PCD inserts was assessed. Similarly, in [17], the chips produced in milling Mg-Ca0.8 alloy under different cutting speeds were analysed. It was noticed that the free surfaces of the chips were characterised by lamella structures. The back surface of the chip is smooth and shiny, which is completely different from the free surface. For example, when observing the shape of the chips [8] produced in AZ31B alloy milling, it was observed that with an increase in the number of inserts in the cutter, the dimensions of the saw-tooth were smaller. The global literature [16] suggests looking for traces of melts or burns on the side surface of chips. SEM scanning electron microscopy was used to identify the melts. No melting points were found on the AZ91 magnesium alloy chips. 

In the work [29], attempts were made to ignite the chips during the machining of AM50A and AZ91D alloys. The following spark types were identified during magnesium alloy milling: minor sparks, major sparks and continuous sparks. The geometry of chips visible in metallographic photos and SEM was also analysed. There are four main chip geometry types: powder chips, long spiral chips, needle-like chips and strip chips. Additionally, the Al-Mn and Mg17Al12 phases were analysed, comparing the microstructure and energy spectrum for the alloy specimens and chips. However, small depths of cut (0.01, 0.04 and 0.08 mm) were used in the experiment. Milled AM50A alloy chips were also researched in [30], where the relationship between the ignition conditions and chip morphologies was analysed. The macromorphologies of the chips were observed by optical microscope and the micro-structures were obtained by scanning electron microscope (SEM). The macromorphologies of the chips can be characterised into powdered chips, tubular helical chips, acicular helical chips and long belt chips, which correspond to the different ignition conditions. The powdered chips and acicular spiral chips are easily ignited and cause flares or continuous flares. These chips have lower lamellar thickness (2–3 μm), compared to tubular helical chips and long belt chips (more than 10 μm). During all of the milling tests of AM50A alloy, the following were observed: sparks, flares and continuous flares. 

It is also possible to assess the quality of the machined surface on the basis of the size and quality of the chips produced, as the appropriate chip geometry results in a high-quality surface. The tests conducted with the use of AZ91D alloy showed that the quality of the chips depends on the feed, while the geometry of the chip is affected by the cutting speed [13].

The works focussed on the analysis of AZ91D/HP [31,32] and AZ31 [33] magnesium alloy chips, including among others the analysis of various types of chip fractions produced during milling. In the works [31,32], tools with different tool point geometries were used (γ_o_ = 5° and γ_o_ = 30°). In the work [33], off-the-shelf TiAlN-coated inserts were used. These works introduce the concept of fraction and fragmentation of chips. In the work [31], chip fragmentation, chip mass and its dimensions are presented. Moreover, in the work [32], the quantity of distinguished chip fractions and the influence of the parameters v_c_ and f_z_, as well as in the rake angle γ_o_ were analysed. It has been found that chip fragmentation increases by increasing the above parameters, i.e., the feed rate f_z_ and the cutting speed v_c_. The observed chip fragmentation (the quantity of chip fractions) is lower at the tool rake angle γ_o_ = 30°. In [23,34], a controlled chip ignition was performed on a specially designed and made chip flammability test stand, outside the machine. The so-called time to ignition of different chip fractions was determined. Additionally, the photos of the stages preceding chip ignition and metallographic photos of chips and ignition products were taken.

Chip classifications can be made based on available standards [35,36]. It should be remembered, though, that these standards only apply to the steel turning. 

### 1.4. Inteligent Methods in Surface and Temperature Parameters Modeling

Increasingly often, experimental research in the field of analysis of machining processes are assisted using machine learning methods, including artificial neural networks [37,38,39]. Based on the analysis of the current literature, one can notice that machine learning is used to model various processes, i.e., milling [40,41,42,43,44,45,46,47,48], turning [37,49,50] and abrasive water jet machining [51,52].

By analysing the data presented in Table 1, it can be seen that in the case of the milling process, the tests were carried out for various materials, such as aluminium alloys [39,43,45,46,47,50,53,54], magnesium alloys [55,56], titanium alloys [37,40,57], cobalt alloys [44], nickel alloys [48,58,59,60] and steel [41,42]. Additionally, it can be stated that as far as the milling roughness parameters are concerned, only 2D parameters are modelled, mainly the Ra parameter. Few researchers have attempted to model more than one parameter. Such works were carried out by, among others Zerti et al., who also modelled the parameters Ra, Rz and Rt, as well as Kulisz et al., who extended their research with the Rz and RSm parameters. It seems that such a narrow scope of research is insufficient to carry out a detailed analysis of surface conditions. In the case of temperature modelling, research works were carried out on materials such as aluminium or nickel alloys [43,53,54,59].

### 1.5. Objective of Research and Novelties

This present work contains an analysis of three machinability indices that directly relate, on the one hand, to the quality of the manufactured elements (3D roughness of the machined surface), and on the other, to the safety of machining (self-ignition hazard—high temperature and geometrical features of chips). The main purpose of the research was to define the machining parameters that ensure the good quality of the surface defined by low 3D roughness parameters, low chip temperature observed in the cutting zone and the characteristics related to the description of the chip fractions (chip geometry) during machining with a HSS tool. Another purpose was to model the 3D parameters of surface roughness (Sa, Sku) and the temperature in the cutting zone after milling AZ91D magnesium alloy in order to predict these parameters.

Taking into account the conducted literature on the machinability of magnesium alloys, it can be concluded that, so far, few researchers have dealt with the 3D roughness parameters for magnesium alloys. They focussed primarily on a 2D parameter—mainly the Ra parameter. There is no wider analysis of a larger group of surface roughness parameters, including a more detailed analysis of the features and operational properties of the machined surface. Therefore, a much broader approach is required to comprehensively assess the quality of the machined surface. Therefore, it is justified to extend the research to a wider range of technological parameters, including 3D; however, one should take into account the tendency of magnesium alloys to self-ignition with a sudden increase in temperature in the cutting zone [30,34]. Additionally, attention should be paid to the characteristics of the chips produced in machining. These features may indicate the safety of the process or the ranges of parameters that should rather be avoided to decrease the risk of ignition.

Modelling the roughness parameters (Sa, Sku) and temperature in the cutting zone in milling AZ91D magnesium alloy can be the basis for creating tools to help manufacturing engineers in determining the conditions of the machining process in order to obtain the required surface roughness and the safety of machining. So far, no such work has been carried out for magnesium alloy machining. In addition, the use of artificial neural networks and other solutions may contribute to the reduction of the number of research tests necessary for the selection and optimisation of the technological process parameters.

Additionally, the analysis may be interesting due to the universality and availability as well as the low cost of the tools themselves, as well as the small amount of research work devoted to milling with the use of HSS milling cutters.

## 2. Materials and Methods

The AZ91D alloy machinability tests were performed on a DMU 65 MonoBlock vertical milling centre 12,000 RPM with the use of many devices and a number of highly specialised scientific and research equipment presented in the further part of the experimental research methodology. The research used a two-teeth HSS Co milling cutter by PRECITOOL with a diameter of d = 20 mm. HSS cutters are widely used tools (lower unit costs), compared to other expensive tools (more expensive carbide tools and very expensive PCD blades, inserts). The study presents the selected parameters of 3D roughness (Sa, Sz, Sv, Sp, Ssk and Sku), the temperature in the cutting zone, and the shape and geometry of chips produced in rough milling. In experimental tasks of individual research works the following measuring equipment was used:

- X6580sc thermal imaging camera from FLIR Systems Inc. (Wilsonville, OR, USA) was used in the thermal imaging tests;
- In the chip geometry tests, SEM technique with an EDS PHENOM ProX electron microscope by ThermoFisher Scientific (Waltham, MA, USA) and an Alicona Infinite Focus microscope (Raaba bei Graz, Austria) were used;
- For the 3D surface roughness measurement, an Alicona Infinite Focus was used.

Thermovision measurements were carried out with the use of the following equipment settings. The camera was located at a distance of 0.6 m from the tested workpiece. The ambient and the cutter temperature were 22 °C before each test. With a resolution of 640 × 512, a frequency of 50 Hz was achieved. The average emissivity of magnesium of 0.13 was taken into account (for the purpose to obtaining the actual temperatures occurring in the milling process).

The milling process was carried out with the following processing conditions: radial depth of cut a_e_ = 15 mm and cutting speed v_c_ = 754 m/min (constant cutting conditions), feed per tooth f_z_ = 0.01–0.05 mm/tooth and axial depth of cut a_p_ = 0.1–0.4 mm (variable technological parameters).

**Figure 2:**
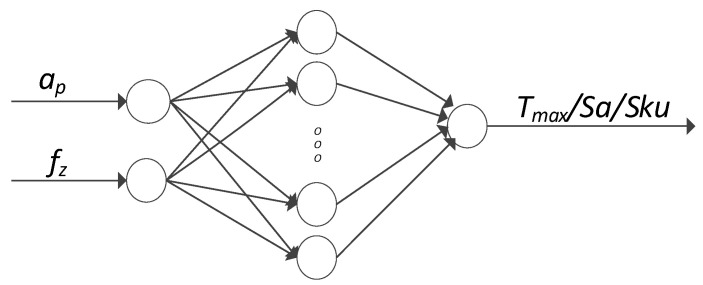
Schematics of the artificial neural network with the analysed process parameters.

Based on the obtained experimental results, attempts were made to model the temperature in the cutting zone and selected 3D roughness parameters—Sa and Sku—with the use of artificial neural networks. Two input neurons were defined in the input layer, i.e., variable processing parameters f_z_ and a_p_. In the output layer, one modelled parameter was defined, i.e., the temperature in the cutting zone, Sa and Sku, respectively. An example of the model is shown in Figure 2. 

Two types of MLP (Multilayer Perceptron) and RBF (Radial Basis Function) networks were used to model the above parameters, the parameters of which are presented in Table 2. They are most often used by researchers when modelling selected indicators of manufacturing processes, which is confirmed by the publications presented in Table 1. The Statistica Neural Networks software was used as the modelling tool.

For each modelled parameter, 200 networks were taught. The network was selected on the basis of the quality of learning and validation, defined as the correlation coefficient for these sets. Learning and validation errors were also taken into account, calculated as the sum of the squared differences between the set values and the values obtained at the outputs of each output neuron, according to the Equation (1):

(1) $$SS=\sum_{i=1}^{n}{\left(y_{i}^{’}-y_{i}^{*}\right)}^{2},$$

where *n* is the number of cases in a given set; $y_{i}^{’}$ is the actual value of the analysed parameter for the given set for the *i*-th observation; and *y*_i_ is the predicted value of the analysed parameter for the given set for the *i*-th observation.

The experimental data set was used in the proportion of 75/25%, where the first value is the share of the teaching data and the second is the share of the validation data.

## 3. Results

### 3.1. Surface 3D Roughness Parameters 

**Figure 3:**
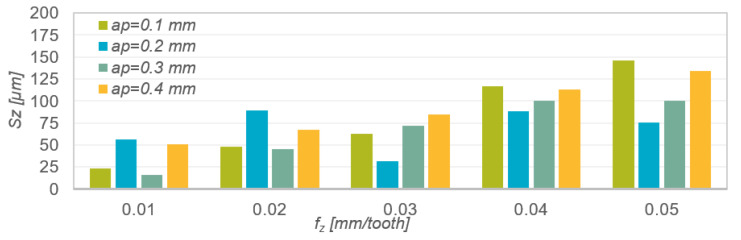
Results of measurements of the Sz roughness parameter as a function of feed per tooth f_z_ and depth of cut a_p_.

The surfaces obtained during machining with variable feed per tooth and cutting depth were analysed based on 3D surface roughness parameters such as Sa, Sz, Sv, Sp, Ssk and Sku. These results were analysed on the face of a sample made of AZ91D alloy. The measurement results are shown in Figure 3.

Table 3 presents the results of mathematical modelling of the spatial surface roughness parameter Sa as a function of the feed per tooth f_z_ for various depths of cut a_p_. The following were selected as regression functions of one variable: linear function, exponential function, logarithmic function, polynomial function and power function. The table shows the coefficient of determination R^2^ and the *p*-value in Fisher’s test of statistical significance of the linear model. If *p*-value is less than 0.05 then R^2^ is significantly different from zero and we assume that the model can be treated as linear. The normality of the rest of the model was checked for each model. The analysis of the modelling results presented in Table 3 shows that the best matches of the experimental results are described by the power function for low depths of cut a_p_ = 0.1 mm (R^2^ = 0.9843) and a_p_ = 0.2 mm (R^2^ = 0.9735), while for higher values of the depth of cut, the best results were achieved for the description of the functional relationships with polynomial regression equations (for a_p_ = 0.3 mm (R^2^= 0.9995) and a_p_ = 0.4 mm (R^2^ = 0.9949)).

Analysis of the Sa parameter (Figure 3) showed that the lowest and also most similar values (Sa = 1.51–1.57 µm) were obtained in machining with the lowest technological parameters. Continued increasing of the feed per tooth *f_z_* resulted in an increase in the value of the Sa parameter. This increase was greater the greater the depth of the cut a_p_ and feed per tooth f_z_. The exception is a_p_ = 0.2 mm, for which the Sa parameter increase was the smallest.

**Figure 4:**
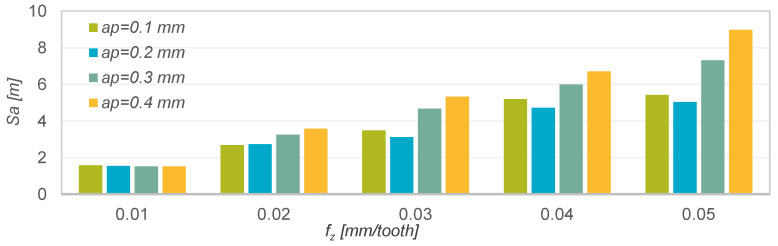
Results of measurements of the Sa roughness parameter as a function of feed per tooth f_z_ and depth of cut a_p_.

As in the case of the Sa parameter, increasing the feed per tooth f_z_ resulted in a gradual increase in the value of parameter Sz (Figure 4). The exceptions are the results obtained when milling with depth of cut a_p_ = 0.2 mm, for which the change of the feed per tooth did not have a clear effect on the parameter Sz. At low feed f_z_, the lowest values of the Sz parameter were obtained at the depths a_p_ = 0.1 and 0.3 mm, while at higher feed rates of f_z_, the lowest roughness height was obtained by using the depths a_p_ = 0.2 and 0.3 mm, respectively.

Table 4 presents the results of mathematical modelling of the spatial surface roughness parameter Sa as a function of the feed per tooth *f_z_*, for various depths of cut a_p_. As the regression functions of one variable, the same function was selected as in the case of the parameter Sa. The table shows the coefficient of determination R^2^ and the *p*-value in Fisher’s test of statistical significance of the linear model. The normality of the rest of the model was checked for each model. The analysis of the modelling results presented in Table 4 shows that the best matches of the results of the experimental research are described by the second-order polynomial function a_p_ = 0.1 mm (R^2^ = 0.9795), a_p_ = 0.2 mm (R^2^ = 0.0709), a_p_ = 0.3 mm (R^2^ = 0.9829) and a_p_ = 0.4 mm (R^2^ = 0.9958).

**Figure 5:**
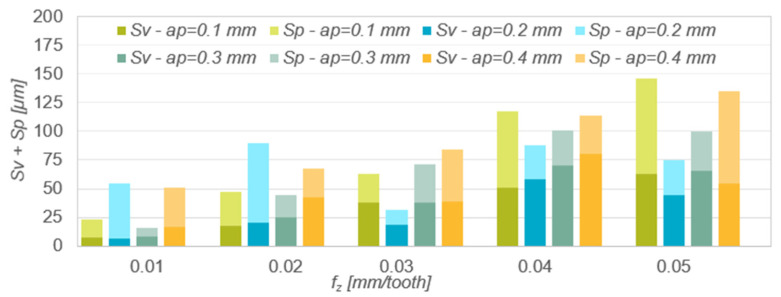
Results of measurements of roughness parameters Sv and Sp as a function of feed per tooth f_z_ and depth of cut a_p_.

Increase in the parameter Sv (Figure 5) in the entire range of feed per tooth occurred only for the smallest depth of cut. For the remaining depths of cut, the size of the recesses increased in the range f_z_ = 0.01–0.04 mm/tooth, and then decreased. In the above-mentioned range of feed per tooth, it was also observed that the smaller the depth of cut was used, the deeper the roughness valleys were. In the case of the Sp parameter, no relationship to the technological parameters variables was observed. The predominance of the roughness valleys over peaks was also random in most cases. In the aforementioned range of feed per tooth, lower values of the parameter Rv were obtained for the lower depths of cut.

Table 5 presents the results of mathematical modelling of the spatial surface roughness parameters Sv and Sp as a function of the feed per tooth f_z_, for various depths of cut a_p_. As regression functions of one variable, the following were selected: the function analysed as in the case of Sa and Sz. The table shows the coefficient of determination R^2^ and the *p*-value in Fisher’s test of statistical significance of the linear model. The normality of the rest of the model was checked for each model. The analysis of the Modelling results presented in Table 5 shows that the best matches of the results of the experimental research are described by the second-order polynomial function a_p_ = 0.1 mm (R^2^ = 0.9139), a_p_ = 0.2 mm (R^2^ = 0.549), a_p_ = 0.3 mm (R^2^ = 0.9465) and a_p_ = 0.4 mm (R^2^ = 0.8089). In the case of the parameter Sp, the best results were achieved for the description of the second-order polynomial functional relationships: a_p_ = 0.1 mm (R^2^ = 0.9896), a_p_ = 0.2 mm (R^2^ = 0.8129), a_p_ = 0.3 mm (R^2^ = 0.9327) and a_p_ = 0.4 mm (R^2^ = 0.769).

**Figure 6:**
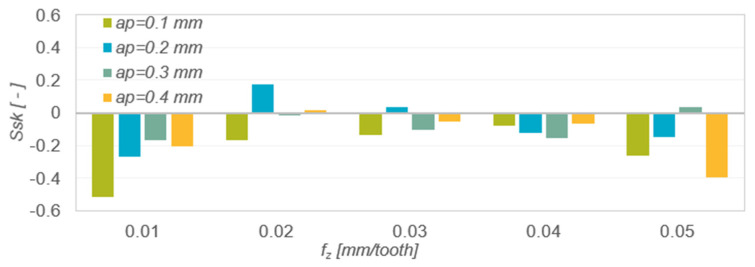
The results of measurements of roughness parameter Ssk as a function of feed per tooth f_z_ and depth of cut a_p_.

In measuring the roughness profile skew (Figure 6) negative values were obtained in most cases. This indicates areas with flat peaks and quite deep individual valleys. This type of surface has a high lubricant retention capacity. The lowest values of the Ssk parameter were obtained when machining with the lowest and highest feed per tooth.

Table 6 presents the results of mathematical modelling of the spatial surface roughness parameter Ssk as a function of the feed per tooth f_z_ for various depths of cut a_p_. The following were selected as regression functions of one variable: linear function, logarithmic function and polynomial function. The table shows the coefficient of determination R^2^ and the *p*-value in Fisher’s test of statistical significance of the linear model. The normality of the rest of the model was checked for each model. The analysis of the modelling results presented in Table 6 shows that the best matches of the results of the experimental research are described by the polynomial function a_p_ = 0.1 mm (R^2^ = 0.9503), a_p_ = 0.2 mm (R^2^ = 0.9823), a_p_ = 0.3 mm (R^2^ = 0.9969) and a_p_ = 0.4 mm (0.9285).

**Figure 7:**
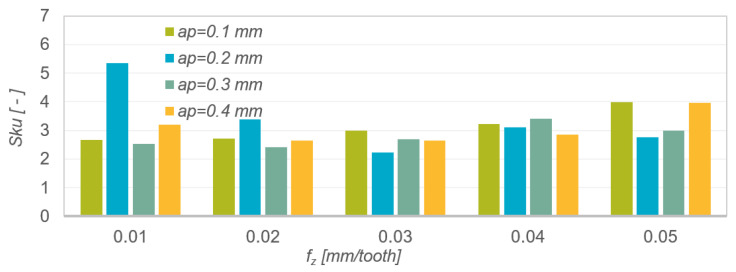
Results of measurements of the roughness parameter Sa as a function of feed per tooth f_z_ and depth of cut a_p_.

The kurtosis value Sku of the roughness profile (Figure 7) in most cases oscillated around 3, which indicates that the roughness distribution was close to normal. The kurtosis value was independent of the change in feed per tooth and depth of cut. Sku values >3 indicate larger roughness valleys and sharper peaks.

Table 7 presents the results of mathematical modelling of the spatial surface roughness parameter Sku as a function of the feed per tooth f_z_ for various depths of cut a_p_. The following were selected as regression functions of one variable: linear function, exponential function, logarithmic function, polynomial function and power function. The table shows the coefficient of determination R^2^ and the *p*-value in Fisher’s test of statistical significance of the linear model. The normality of the rest of the model was checked for each model. The analysis of the modelling results presented in Table 7 shows that the best matches of the results of the experimental research are described by the polynomial function a_p_ = 0.1 mm (R^2^ = 0.9847), a_p_ = 0.2 mm (R^2^ = 0.9513), a_p_ = 0.3 mm (R^2^ = 0.9416) and a_p_ = 0.4 mm (0.9864).

### 3.2. Thermovision Tests—Temperature of Chips Produced during AZ91D Magnesium Alloy Milling

**Figure 8:**
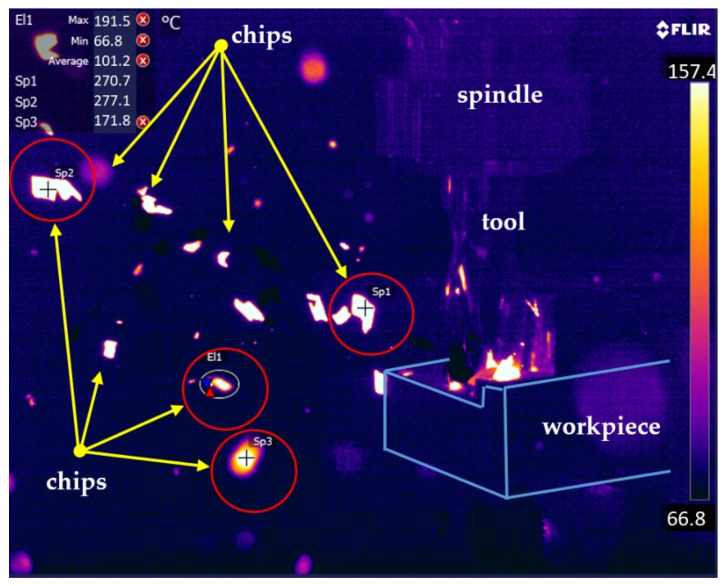
Exemplary thermal imaging of chips removed from the working space during machining.

Figure 8 presents an example of a thermal image recorded during the milling of the AZ91D alloy with an HSS tool with marked areas of chip temperature measurement. The characteristic chips, along with the recorded values of their temperature, are marked.

**Figure 9:**
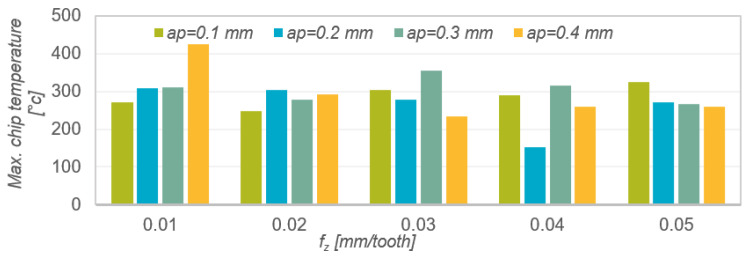
Results of measurements of the chips’ maximum temperature as a function of feed per tooth and depth of cut.

The results of the maximum chip temperature measurement as a function are presented in the form of a graph in Figure 9. When analysing the obtained results, no clear correlation was observed between the change in feed per tooth and depth of cut, and the maximum temperature. The obtained temperature values oscillated around 300 °C. The differences in the obtained results may be largely due to the fact that the chip formation process is a rapidly changing phenomenon.

Table 8 presents the results of mathematical modelling of the spatial surface roughness parameter Sku as a function of the feed per tooth f_z_ for various depths of cut a_p_. The following were selected as regression functions of one variable: linear function, exponential function, logarithmic function, polynomial function and power function. The table shows the coefficient of determination R^2^ and the *p*-value in Fisher’s test of statistical significance of the linear model. The normality of the rest of the model was checked for each model. The analysis of the modelling results presented in Table 7 shows that the best matches of the results of the experimental research are described by the polynomial function a_p_ = 0.1 mm (R^2^ = 0.7074), a_p_ = 0.2 mm (R^2^ = 0.8483), a_p_ = 0.3 mm (R^2^ = 0.658) and a_p_ = 0.4 mm (0.9907).

Modelling with regression functions was aimed at selecting the optimal mathematical solution describing the variability of the output parameters of the model with the input data. The coefficients of the studied regression functions were changed so that the value of the coefficient of determination R^2^ was as high as possible. The high value of R^2^ is a measure of what percentage of the variability of the dependent variable (f_z_) is explained by the independent variable (modelled parameter).

### 3.3. AZ91D Magnesium Alloy Chip Geometry

**Figure 10:**
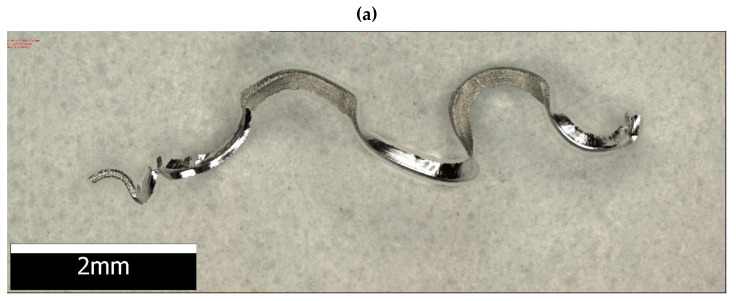

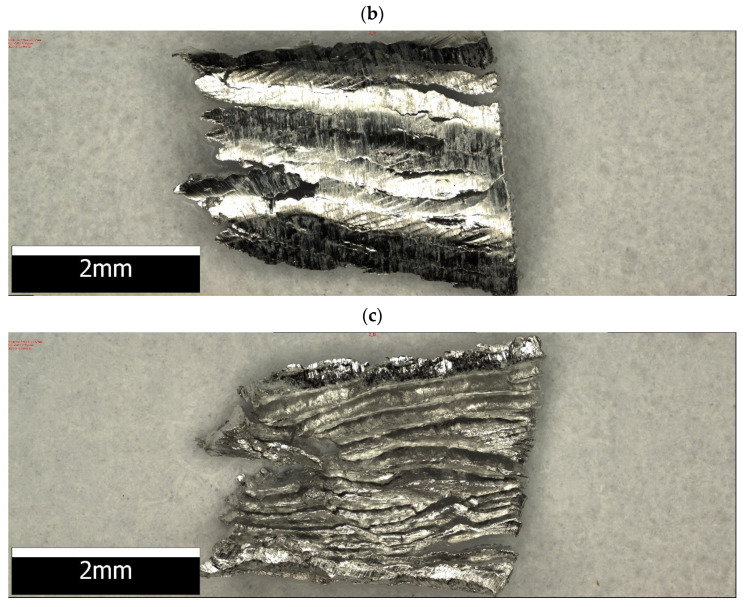
Optical imaging of chip geometry: (**a**) general view, (**b**) rake face side view and (**c**) free side view.

Figure 10a presents an example of an optical representation of a magnesium alloy chip after milling, Figure 10b presents an enlarged visual image from the face side, and Figure 10c presents an enlarged visual image from the free side. The presented images show a relatively smooth surface of the chip with visible cracks in the transverse direction. There are no visible traces of burns and melting marks indicating the possibility of uncontrolled ignition in machining. The free chip flow side has numerous shifts in the slip planes that look like a stepped structure. The free surface on the chip flow on the rake face has a reflective smooth surface with numerous discontinuities of the structure damaged as a result of force interactions, stresses and shear in the sliding planes.

The chips shown in Figure 10a can be classified as so-called snarled chips (long or entangled). This is the type of chips that should be avoided due to their shape. In contrast, the chips shown in Figure 10b,c are chips classified as so-called arc loose, and this is the preferred type of chips. Nevertheless, all of the types of chips outlined above are easily removed from the cutting zone and are generally not very hazardous due to their shape.

**Figure 11:**
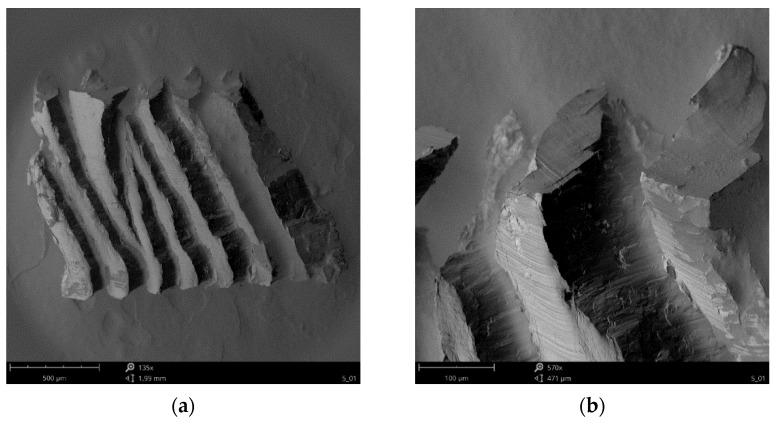
SEM imaging of a magnesium alloy chip surface on the free side (**a**) slip plane and (**b**) material decohesion.

**Figure 12:**
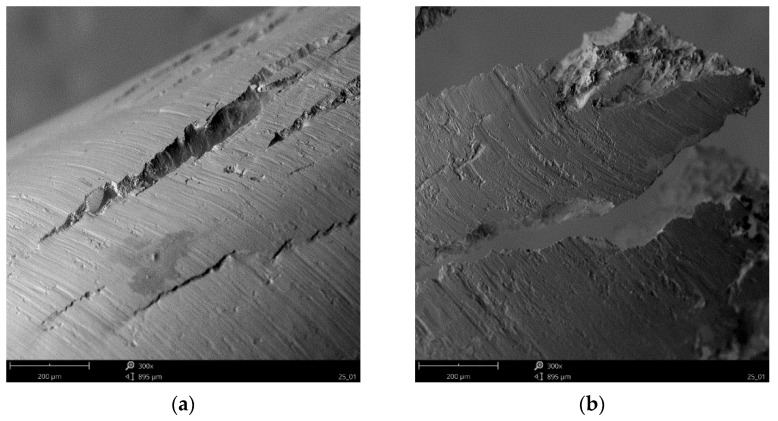
SEM imaging of a magnesium alloy chip surface from the chip flow side on the rake face (**a**) cracks and (**b**) tearing on the free edges.

Whereas in Figure 11 and Figure 12 SEM photos of chips produces during the milling of AZ91D alloy are presented. The high quality of the observed chip surfaces in this case also have no traces of any burn marks or melting. This may mean that the milling is still safe and there is no risk of uncontrolled ignition when machining with the pre-set technological parameters. On the free surface of the chip, characteristic slip planes are visible (Figure 11a) and material decohesion (Figure 11b). The structure of the free surface is “ball-shaped” with clearly visible “steps”. On the chip flow side, on the rake face, the chip surface is relatively flat, with visible traces in the form of scratches parallel to the chip flow velocity vector and cracks (Figure 12a) and tearing (defragmentation) (Figure 12b).

### 3.4. Artificial Neural Network Simulation

The results of the modelling performed together with the parameters of the networks are presented in Table 3. One network of each type (RBG, MLP) was selected for the analysed roughness parameters (Sa and Sku) and the maximum temperature under cutting stress. These networks were selected on the basis of network errors and quality of learning and validation. The best parameters for maximum temperature were obtained for the RBF 2-13-1 network with thirteen neurons in the hidden layer, and in the case of roughness parameters—for the MLP network. For the Sa parameter, it is a network with four neurons in the hidden layer (MLP 2-4-1), created in 8654 iterations, whereas for the SKu parameter, with 5 neurons (MLP 2-5-1), created in 4659 iterations. The quality of both learning and validation for these networks exceeds 0.95. Additionally, Table 9 contains the correlation coefficients R, which, when analysed, showed that the interdependence between the experimental data and those predicted for these selected networks is at a high level (above 0.94).

**Figure 13:**
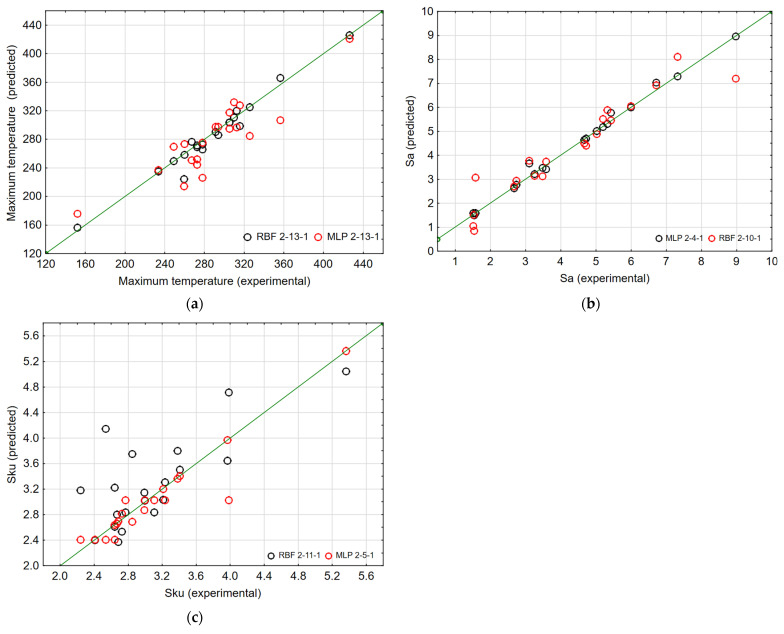
Correlation graph of comparison between the modelling and experimental results of the RBF and MPL networks for (**a**) Maximum temperature, (**b**) Sa parameter and (**c**) Sku parameter.

The comparison of the results obtained as a result of RBF and MLP network modelling presented in Table 9 are also featured in Figure 13, where the correlation of the individual analysed parameters obtained experimentally and those obtained as a result of modelling can be seen. The figures confirm that for the maximum temperature, better matched results were obtained using the RBF network, whereas for Sa and Sku, using the MLP network.

**Figure 14:**
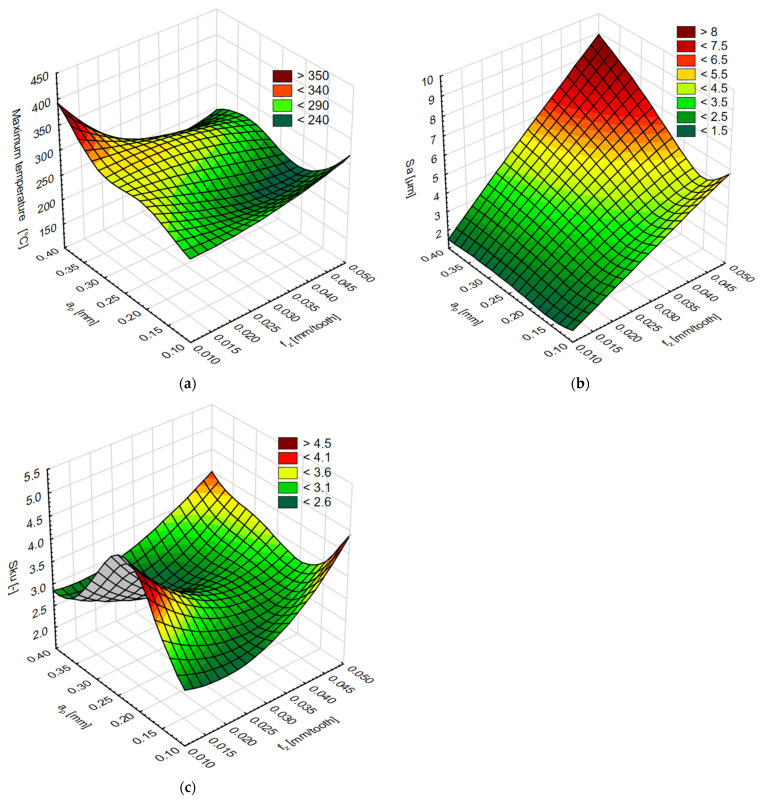
The simulation results of the variable feed per tooth f_z_ and depth of cut a_p_ for (**a**) maximum temperature, (**b**) Sa parameter and (**c**) Sku parameter.

As a result of the modelling procedure, it was possible to predict the maximum temperature and the Sa and Sku parameters with the use of selected networks. After entering the new data into the Statistica software (feed per tooth and milling depth, respectively), the predicted values of the analysed parameters were generated. The results of the network operation are presented for the RBF 2-13-1 network (maximum temperature) in Figure 14a, for the MLP 2-4-1 network (parameter Sa) in Figure 14b, and for MLP 2-5-1 (parameter Sku)—on Figure 14c.

In order to determine whether each of the input technological parameters affects the maximum temperature and the Sa/Sku parameters, a sensitivity analysis was performed (Table 10). None of the analysed technological parameters obtained a sensitivity analysis value below 1, which means that each of them had a significant impact. The parameter that had the most significant influence on the maximum temperature and the Sa and Sku parameters is feed per tooth.

As a result of modelling the maximum temperature and Sa and Sku parameters, as well as the prediction made, it can be concluded that the obtained RBF and MLP networks have a satisfactory ability to predict these parameters. This is confirmed by, among others, the R correlation value at the level of 0.94, high quality of learning and network validation at the level of 0.95, and learning and validation errors. Comparing the experimental and simulation data of the individual analysed parameter values, it can be concluded that the value of the relative error does not exceed 15%, which proves that the network is well trained.

The black box model (built on the basis of artificial neural networks) was assessed on the basis of quality of learning and validation as well as learning and validation errors. Based on the simulations performed, the best networks describing the modelled relationships were determined. The results of the network fit and the correlation of the modelled relationships were also described using the correlation coefficients R. The simulations show that the best network describing the relationship between the dependent variable (Sa) and independent variables (f_z_ and a_p_) is MLP 2-4-1, for which R_Sa_ = 0.9970, for the Sku variable—MLP 2-5-1 network, for which R_Sku_ = 0.9414, and for the Maximum temperature variable—RBF 2-13-1 network, for which R_maxT_ = 0.9835.

## 4. Conclusions

Based on the conducted research, the following conclusions can be drawn:

- An increase of the feed per tooth f_z_ in most cases resulted in an increase in surface roughness parameters Sa, Sz and Sv. Higher values of these roughness parameters were recorded for greater depths of cut ap. For parameters Sp, Ssk and Sku, no clear relationships were observed with regard to the change in machining conditions. The results of mathematical modelling proved that the best matching to the values of the resulting surface geometric structure parameters was obtained for the regression function in the form of a second and third degree polynomial. The obtained values of the coefficient of determination R^2^ for the built models were in the range 0.5490–0.9995, except for Sz for a_p_ = 0.2, for which R^2^ was 0.0709. It should be noted, however, that for the majority of the developed models, the value of the coefficient of determination R^2^ is higher than 0.80 and only a few models have lower values of the coefficient of determination;
- Changing the feed per tooth f_z_ and the depth of cut ap in the analysed ranges did not have a significant effect on the maximum temperature of the chips produced in milling (T);
- In most cases, the temperature of the chip observed during milling was around 300 °C, which is considered to be a safe chip temperature in terms of self-ignition hazard;
- The presented metallographic photos of chips, as well as the imaging performed using a SEM, make it possible to conclude that the milling process is safe (no burn marks or chip melting);
- The presented selected representations of chips belong to different groups of chips, both snarled chips and loose chips, which are more favourable due to their shape;
- For modelling the maximum temperature obtained in milling AZ91D magnesium alloy with the use of a HSS tool, the RBF neural network was found to be a better type of network than MLP. For the RBF network, compared to MLP, the quality of learning and validation is higher, and the errors are less significant;
- In the case of the 3D roughness parameters, a better result was obtained for the MLP network;
- The obtained results of the network modelling show a satisfactory predictive ability, as evidenced by the obtained values of correlation R. The values are R_maxT_ = 0.98353, R_Sa_ = 0.997018 and R_Sku_ = 0.941437, respectively. Therefore, it can be concluded that artificial neural networks are effective tools for predicting these parameters. Based on the comparative assessment of the parameters of the mathematical models and those made with the use of artificial neural networks, it can be indicated that 8 ÷ 14% of models based on artificial intelligence show better matching results than most polynomial mathematical models;
- Modelling of processes can constitute the basis for creating tools that are helpful in the work of manufacturing engineers when determining the conditions of the machining process, in order to obtain the required surface roughness and to maintain safe machining parameters. In addition, it can save time and effort and eliminate costs that would have to be incurred in the case of further machining tests.

## Figures and Tables

**Table 1:** Comparison of modelling methods using the ANN for the milling.

Type of Machining	Research Object	Methods *	Material	Year	Reference
milling	Ra	ANN	Ti–6Al–4V	2016	[40]
milling	Ra, T	ANFIS, ANN	Inconel 690	2017	[58]
milling	Sa	ANN, GA, RSM	DD5	2018	[48]
milling	T	ANN-GA	AA6061 T6	2018	[53]
milling	T	ANN	Inconel 718	2018	[59]
milling	Ra	ANN-GA	AZ91D	2018	[56]
milling	Ra	ANN	S45C steel	2019	[41]
milling	Ra	ANN	Ti-6Al-4V	2019	[57]
milling	Ra, T	ANN-GA	AISI D3	2019	[43]
milling	Ra	ANFIS, ANN-GA	AA6061, AA2024, AA7075	2019	[46]
milling	Ra	ANN, SVM, RA	AA 075-T6	2019	[47]
milling	Ra, Rz	ANN	Inconel 718	2020	[60]
milling	Ra	ANN-GA	P1.2738	2020	[42]
milling	T, Ra	ANN, FL, GA	AA7075	2020	[54]
milling	Ra	ANN	AA6061	2021	[45]
milling	Ra, Rz, RSm	ANN	AZ91D	2021	[60]
dry milling	Ra	ANN	Co–28Cr–6Mo, Co–20Cr–15W–10Ni	2021	[44]
dry turning	Ra, Rz, Rt	ANN	AISI420	2019	[39]
low speed turning	Ra	ANN	AISI316	2015	[50]

* artificial neural network—ANN, adaptive neuro-fuzzy inference system—ANFIS, fuzzy logic—FL, genetic algorithm—GA, regression analysis—RA; response surface method—RSM, temperature—T.

**Table 2:** Artificial neural network learning parameters.

ANN Types	Activation Function	Learning Algorithm	Hidden-Layer Neurons	Training Epochs
MLP	exponential, logistic, linear, tanh and sinus	BFGS	2÷15	150–300
RBF	Gaussian, linear	RBFT		

**Table 3:** Results of mathematical modelling of the Sa parameter as a function of the feed rate for different values of the depth of cut (regression functions y = Sa(f_z_); f_z_ = x with the coefficient of determination R^2^).

**a**_**p**_ ** = 0.1 mm**	y = 1.0213x + 0.6017 R^2^ = 0.9652 (*p* = 0.0028)	**a**_**p**_ ** = 0.2 mm**	y = 0.8945x + 0.7405 R^2^ = 0.9596 (*p* = 0.0035)	**a**_**p**_ ** = 0.3 mm**	y = 1.4353x + 0.2389 R^2^ = 0.9966 (*p* = 0.0001)	**a**_**p**_ ** = 0.4 mm**	y = 1.803x − 0.1902 R^2^ = 0.9949 (*p* = 0.0002)
	y = 1.2984e^0.3141x^ R^2^ = 0.9401 (*p* = 0.0063)		y = 1.3174e^0.2906x^ R^2^ = 0.9287 (*p* = 0.0083)		y = 1.2856e^0.3769x^ R^2^ = 0.9199 (*p* = 0.0099)		y = 1.2673e^0.4184x^ R^2^ = 0.9215 (*p* = 0.0096)
	y = 2.4998ln(x) + 1.2720 R^2^ = 0.9341 (*p* = 0.0073)		y = 2.1884ln(x) + 1.3286; R^2^ = 0.9279 (*p* = 0.0084)		y = 3.5233ln(x) + 1.1713; R^2^ = 0.9701 (*p* = 0.0022)		y = 4.38ln(x) + 1.0249 R^2^ = 0.9485 (*p* = 0.0050)
	y = −0.0601x^2^ + 1.3817x + 0.1812; R^2^ = 0.9698 (*p* = 0.0302)		y = −0.0371x^2^ + 1.1169x + 0.481; R^2^ = 0.9619 (*p*= 0.0381)		**y = −0.0661x**^**2**^** + 1.8317x − 0.2236;** **R**^**2**^** = 0.9995 **(*p* = 0.0005)		**y = 0.002x**^**2**^** + 1.791x − 0.1762;** **R**^**2**^** = 0.9949 **(*p* = 0.0051)
	**y = 1.5492x**^**0.7996**^ R^**2**^** = 0.9843 **(*p*= 0.0008)		**y = 1.5507x**^**0.7403**^ R^**2**^** = 0.9735 **(*p* = 0.0018)		y = 1.5642x^0.9759^ R^2^ = 0.9965 (*p* = 0.0001)		y = 1.5785x^1.0815^ R^2^ = 0.9947 (*p* = 0.0002)

**Table 4:** Results of mathematical modelling of the Sz parameter as a function of the feed rate for different values of the depth of cut (regression functions y = Sa(f_z_); f_z_ = x with the coefficient of determination R^2^).

**a_p_ = 0.1 mm**	y = 31.469x − 15.111 R^2^ = 0.9627 (*p* = 0.0031)	**a_p_ = 0.2 mm**	y = 3.656x + 57.136 R^2^ = 0.0558 (*p* = 0.7021)	**a_p_ = 0.3 mm**	y = 22.381x − 0.641 R^2^ = 0.9393 (*p* = 0.0065)	**a_p_ = 0.4 mm**	y = 21.313x + 26.095 R^2^ = 0.989 (*p* = 0.0005)
	y = 16.603e^0.4565x^ R^2^ = 0.9588 (*p* = 0.0021)		y = 53.741e^0.0566x^ R^2^ = 0.0577 (*p* = 0.7409)		y = 14.293e^0.4498x^ R^2^ = 0.7779 (*p* = 0.0268)		y = 40.48^e0.2466x^ R^2^ = 0.9911 (*p* = 0.0002)
	y = 73.803ln(x) + 8.6294 R^2^ = 0.8555 (*p* = 0.0244)		y = 8.5086ln(x) + 59.957; R^2^ = 0.0488 (*p* = 0.7210)		y = 56.532ln(x) + 12.373; R^2^ = 0.9681 (*p* = 0.0024)		y = 50.547ln(x) + 41.64 R^2^ = 0.8986 (*p* = 0.0141)
	**y = 3.505x**^**2**^** + 10.439x + 9.424;** **R**^**2**^** = 0.9795 **(*p* = 0.0205)		**y = 1.6114x**^**2**^** − 6.0126x + 68.416;** **R**^**2**^** = 0.0709 **(*p* = 0.9291)		**y = −4.0779x**^**2**^** + 46.848x − 29.186;** **R**^**2**^** = 0.9829 **(*p* = 0.0171)		**y = 1.4964x**^**2**^** + 12.334x + 36.57;** **R**^**2**^** = 0.9958 **(*p* = 0.0042)
	y = 21.99x^1.1368^ R^2^ = 0.9696 (*p* = 0.0019)		y = 57.338x^0.1098^ R^2^ = 0.0493 (*p* = 0.7975)		y = 17.507x^1.1974^ R^2^ = 0.9215 (*p* = 0.0023)		y = 47.588x^0.6037^ R^2^ = 0.9658 (*p* = 0.0031)

**Table 5:** Results of mathematical modelling of the Sv and Sp parameters as a function of the feed rate for various values of the depth of cut (regression functions y = Sv(f_z_), y = Sp(f_z_); f_z_ = x with the coefficient of determination R^2^).

**Sp**	**a**_**p**_ ** = 0.1 mm**	y = 16.988x − 7.19 R^2^ = 0.8554 (*p* = 0.0005)	**a**_**p**_ ** = 0.2 mm**	y = −7.399x + 60.241 R^2^ = 0.3027 (*p* = 0.0651)	**a**_**p**_ ** = 0.3 mm**	y = 6.3234x + 5.8894 R^2^ = 0.7888 (*p* = 0.0101)	**a**_**p**_ ** = 0.4 mm**	y = 9.928x + 13.612 R^2^ = 0.5327 (*p* = 0.1232)
		y = 10.618e^0.4095x^ R^2^ = 0.9093 (*p* = 0.0091)		y = 55.657e^−0.174x^ R^2^ = 0.319 (*p* = 0.0374)		y = 7.7707e^0.3446x^ R^2^ = 0.6324 (*p* = 0.0192)		y = 21.924e^0.1994x^ R^2^ = 0.613 (*p* = 0.0883)
		y = 38.745ln(x) + 6.6755 R^2^ = 0.7188 (*p* = 0.0069)		y = −18.31ln(x) + 55.573; R^2^ = 0.2994 (*p* = 0.0752)		y = 16.909ln(x) + 8.6697; R^2^ = 0.9112 (*p* = 0.0131)		y = 20.619ln(x) + 23.65 R^2^ = 0.3712 (*p* = 0.0897)
		**y = 3.7557x**^**2**^** − 5.5463x + 19.1;** **R**^**2**^** = 0.9139 **(*p* = 0.0104)		**y = 5.0942x**^**3**^** − 43.585x**^**2**^** + 99.25x − 9.506;** **R**^**2**^** = 0.549 **(*p* = 0.5331)		**y = −2.3896x**^**2**^** + 20.661x − 10.838;** **R**^**2**^** = 0.9465 **(*p* = 0.0673)		**y = 2.3158x**^**3**^** − 15.277x**^**2**^** + 31.187x + 13.666;** **R**^**2**^** = 0.8089 **(*p* = 0.5875)
		y = 14.218x^0.9781^ R^2^ = 0.8532 (*p* = 0.0004)		y = 52.314x^−0.481^ R^2^ = 0.2735 (*p* = 0.0205)		y = 8.7915x^0.9507^ R^2^ = 0.7964 (*p* = 0.0019)		y = 26.84x^0.4134^ R^2^ = 0.4399 (*p* = 0.0400)
**Sv**	**a**_**p**_ ** = 0.1 mm**	y = 14.478 x − 7.9082 R^2^ = 0.9891 (*p* = 0.0244)	**a**_**p**_ ** = 0.2 mm**	y = 11.456x − 4.7116 R^2^ = 0.7304 (*p* = 0.3366)	**a**_**p**_ ** = 0.3 mm**	y = 16.058x − 6.5352 R^2^ = 0.9185 (*p* = 0.0441)	**a**_**p**_ ** = 0.4 mm**	y = 11.395x + 12.461 R^2^ = 0.6015 (*p* = 0.1615)
		y = 5.6101e^0.5324x^ R^2^ = 0.8885 (*p* = 0.0209)		y = 5.1903e^0.4945x^ R^2^ = 0.6185 (*p* = 0.4574)		y = 6.9099e^0.5178x^ R^2^ = 0.7673 (*p* = 0.0654)		y = 16.759e^0.3015x^ R^2^ = 0.4937 (*p* = 0.1795)
		y = 35.053ln(x) + 1.9623 R^2^ = 0.9367 (*p* = 0.0696)		y = 28.004ln(x) + 2.8435; R^2^ = 0.7051 (*p* = 0.3398)		y = 39.623ln(x) + 3.6995; R^2^ = 0.9035 (*p* = 0.0116)		y = 29.949ln(x) + 17.97 R^2^ = 0.6713 (*p* = 0.2754)
		**y = −0.2556x**^**2**^** + 16.011x − 9.6972;** **R**^**2**^** = 0.9896 **(*p*= 0.0861)		**y = −3.0723x**^**3**^** + 26.715x**^**2**^** − 55.435x + 40.352;** **R**^**2**^** = 0.8129 **(*p* = 0.7855)		**y = −1.687x**^**2**^** + 26.18x − 18.344;** **R**^**2**^** = 0.9327 **(*p* = 0.0535)		**y = −3.0083x**^**3**^** + 23.011x**^**2**^** − 35.22x + 34.556;** **R**^**2**^** = 0.769 **(*p* = 0.5384)
		y = 7.4551x^1.371^ R^2^ = 0.9808 (*p* = 0.0401)		y = 6.7438x^1.2758^ R^2^ = 0.7217 (*p* = 0.4027)		y = 8.9054x^1.3574^ R^2^ = 0.8982 (*p* = 0.0145)		y = 18.922x^0.8178^ R^2^ = 0.6194 (*p* = 0.2936)

**Table 6:** Results of mathematical modelling of the Ssk parameter as a function of the feed rate for different values of the depth of cut (regression functions y = Ssk(f_z_); f_z_ = x with the coefficient of determination R^2^).

**a**_**p**_ ** = 0.1 mm**	y = 0.0587x − 0.4083 R^2^ = 0.2931 (*p* = 0.3460)	**a**_**p**_ ** = 0.2 mm**	y = −0.0065x − 0.0471 R^2^ = 0.0035 (*p* = 0.9246)	**a**_**p**_ ** = 0.3 mm**	y = 0.0261x − 0.1609 R^2^ = 0.2184 (*p* = 0.4274)	**a**_**p**_ ** = 0.4 mm**	y = −0.0459x − 0.003 R^2^ = 0.1936 (*p* = 0.4584)
	y = 0.1922ln(x) − 0.4164 R^2^ = 0.5082 (*p* = 0.1765)		y = 0.0394ln(x) − 0.1043; R^2^ = 0.021 (*p* = 0.8163)		y = 0.0645ln(x) − 0.1445; R^2^ = 0.2161 (*p* = 0.4301)		y = −0.061ln(x) − 0.0821 R^2^ = 0.0556 (*p* = 0.7026)
	**y = −0.0743x**^**2**^** + 0.5042x − 0.9281;** **R**^**2**^** = 0.9503 **(*p* = 0.0497)		**y = 0.0599x**^**3**^** − 0.6076x**^**2**^** + 1.818x − 1.5327;** **R**^**2**^** = 0.9823 **(*p* = 0.1688)		**y = 0.0402x**^**3**^** − 0.3539x**^**2**^** + 0.9263x − 0.7794;** **R**^**2**^** = 0.9969 **(*p*= 0.0711)		**y = −0.0756x**^**2**^** + 0.4077x − 5323;** **R**^**2**^** = 0.9285 **(*p*= 0.0715)

**Table 7:** Results of mathematical modelling of the Sku parameter as a function of the feed rate for different values of the depth of cut (regression functions y = Sku(f_z_); f_z_ = x with the coefficient of determination R^2^).

**a**_**p**_ ** = 0.1 mm**	y = 0.3139x + 2.1769 R^2^ = 0.8684 (*p* = 0.0211)	**a**_**p**_ ** = 0.2 mm**	y = −0.5463x + 5.0093 R^2^ = 0.5257 (*p* = 0.1657)	**a**_**p**_ ** = 0.3 mm**	y = 0.1913x + 2.2297 R^2^ = 0.5682 (*p* = 0.1411)	**a**_**p**_ ** = 0.4 mm**	y = 0.1723x + 2.5429 R^2^ = 0.2397 (*p* = 0.4025)
	y = 2.3037e^0.0973x^ R^2^ = 0.9026 (*p* = 0.0135)		y = 4.9188e^−0.141x^ R^2^ = 0.5971 (*p* = 0.1994)		y = 2.2689e^0.0679x^ R^2^ = 0.5636 (*p* = 0.1262)		y = 2.6017e^0.05x^ R^2^ = 0.265 (*p* = 0.4340)
	y = 0.6998ln(x) + 2.4486 R^2^ = 0.6972 (*p* = 0.0784)		y = −1.594ln(x) + 4.8968; R^2^ = 0.7231 (*p* = 0.0679)		y = 0.4503ln(x) + 2.3725 R^2^ = 0.5085 (*p* = 0.1763)		y = 0.2537ln(x) + 2.817 R^2^ = 0.0839 (*p* = 0.6363)
	**y = 0.0971x**^**2**^** − 0.2685x + 2.8564;** **R**^**2**^** = 0.9847 **(*p* = 0.0497)		**y = −0.1695x**^**3**^** + 1.9037x**^**2**^** − 6.8158x + 10.504;** **R**^**2**^** = 0.9513 **(*p* = 0.2786)		**y = −0.1288x**^**3**^** + 1.1493x**^**2**^** − 2.7879x + 4.3226;** **R**^**2**^** = 0.9416 **(*p* = 0.3046)		**y = 0.0287x**^**3**^** − 0.0026x**^**2**^** − 0.6833x + 3.8488;** **R**^**2**^** = 0.9864 **(*p* = 0.1479)
	y = 2.5003x^0.2194^ R^2^ = 0.7397 (*p* = 0.0613)		y = 4.787x^−0.413^ R^2^ = 0.7903 (*p* = 0.0963)		y = 2.3886x^0.1591^ R^2^ = 0.5245 (*p* = 0.1641)		y = 2.8297x^0.0689^ R^2^ = 0.0923 (*p* = 0.6781)

**Table 8:** Results of mathematical modelling of the T parameter as a function of the feed rate for different values of the depth of cut (regression functions y = T(f_z_); f_z_ = x with the coefficient of determination R^2^).

**a**_**p**_ ** = 0.1 mm**	y = 14.89x + 243.97 R^2^ = 0.6384 (*p* = 0.1049)	**a**_**p**_ ** = 0.2 mm**	y = −22.72x + 331.38 R^2^ = 0.3128 (*p* = 0.3270)	**a**_**p**_ ** = 0.3 mm**	y = −5.24x + 321.4 R^2^ = 0.0551 (*p* = 0.7039)	**a**_**p**_ ** = 0.4 mm**	y = −36.7x + 404.56 R^2^ = 0.5737 (*p* = 0.1381)
	y = 246.33e^0.0514x^ R^2^ = 0.6477 (*p* = 0.1137)		y = 339.61e^−0.095x^ R^2^ = 0.3305 (*p* = 0.3797)		y = 321.38e^−0.018x^ R^2^ = 0.0523 (*p* = 0.6788)		y = 401.76e^−0.111x^ R^2^ = 0.6345 (*p* = 0.1424)
	y = 33.236ln(x) + 256.82 R^2^ = 0.5138 (*p* = 0.1730)		y = −58.81ln(x) + 319.53; R^2^ = 0.3386 (*p* = 0.3033)		y = −7.793ln(x) + 313.14 R^2^ = 0.0197 (*p* = 0.8219)		y = −106.5ln(x) + 396.48 R^2^ = 0.7811 (*p* = 0.0467)
	**y = −2.525x**^**3**^** + 25.975x**^**2**^** − 64.2x + 309.14;** **R**^**2**^** = 0.7074 **(*p* = 0.6535)		**y = 22.367x**^**3**^** − 190.5x**^**2**^** + 440.33x + 31.22;** **R**^**2**^** = 0.8483 **(*p* = 0.4831)		**y = −10.017x**^**3**^** + 79.593x**^**2**^** − 178.29x + 415.78;** **R**^**2**^** = 0.658 **(*p* = 0.6996)		**y = −8.125x**^**3**^** + 98.254x**^**2**^** − 379.22x + 716.96;** **R**^**2**^** = 0.9907 **(*p* = 0.1227)
	y = 257.55x^0.1146^ R^2^ = 0.5317 (*p* = 0.1831)		y = 323.46x^−0.247^ R^2^ = 0.3342 (*p* = 0.3550)		y = 312.75x^−0.029^ R^2^ = 0.0191 (*p* = 0.7930)		y = 391.91x^−0.323^ R^2^ = 0.8364 (*p* = 0.0508)

**Table 9:** Selected networks based on quality (learning, validation), errors (learning, validation).

Network Name	Quality (Training)	Quality (Validation)	SS (Training)	SS (Validation)	Activation (Hidden)	Activation (Output)	R_(i)_ Correlation
Maximum Temperature							
**RBF** 2-13-1	0.9947	0.9837	17.3924	143.9912	Gaussian	Linear	0.9835
**MLP** 2-13-1	0.9377	0.8392	202.8053	652.0954	Tanh	Sinus	0.8917
**Sa**							
**RBF** 2-10-1	0.9432	0.9849	0.2431	0.0726	Gaussian	Linear	0.9506
**MLP** 2-4-1	0.9999	0.9924	0.0019	0.0565	Logistic	Linear	0.9970
**Sku**							
**RBF** 2-11-1	0.9287	0.7085	0.0335	0.4862	Gaussian	Linear	0.7440
**MLP** 2-5-1	0.9903	0.9538	0.0046	0.1025	Tanh	Exponential	0.9414

**Table 10:** Sensitivity analysis values for the technological parameters: feed per tooth f_z_ and axial depth of cut a_p_.

Sensitivity Analysis		f_z_	a_p_
Maximum temperature	RBF 2-13-1	43.6506	28.7312
Sa	MLP 2-4-1	127.9466	32.3221
Sku	MLP 2-5-1	445.2054	12.2694

## Data Availability

Not applicable.
